# The Role of Cardiovascular Imaging in the Diagnosis of Athlete’s Heart: Navigating the Shades of Grey

**DOI:** 10.3390/jimaging10090230

**Published:** 2024-09-14

**Authors:** Nima Baba Ali, Sogol Attaripour Esfahani, Isabel G. Scalia, Juan M. Farina, Milagros Pereyra, Timothy Barry, Steven J. Lester, Said Alsidawi, David E. Steidley, Chadi Ayoub, Stefano Palermi, Reza Arsanjani

**Affiliations:** 1 Department of Cardiovascular Medicine, Mayo Clinic, Phoenix, AZ 85054, USA; 2 Public Health Department, University of Naples Federico II, via Pansini 5, 80131 Naples, Italy; stefano.palermi@unina.it

**Keywords:** athlete’s heart, cardiac computed tomography, cardiac magnetic resonance, cardiomyopathies, echocardiography, strain, stress echocardiography, artificial intelligence, deep learning, sudden cardiac death

## Abstract

Athlete’s heart (AH) represents the heart’s remarkable ability to adapt structurally and functionally to prolonged and intensive athletic training. Characterized by increased left ventricular (LV) wall thickness, enlarged cardiac chambers, and augmented cardiac mass, AH typically maintains or enhances systolic and diastolic functions. Despite the positive health implications, these adaptations can obscure the difference between benign physiological changes and early manifestations of cardiac pathologies such as dilated cardiomyopathy (DCM), hypertrophic cardiomyopathy (HCM), and arrhythmogenic cardiomyopathy (ACM). This article reviews the imaging characteristics of AH across various modalities, emphasizing echocardiography, cardiac magnetic resonance (CMR), and cardiac computed tomography as primary tools for evaluating cardiac function and distinguishing physiological adaptations from pathological conditions. The findings highlight the need for precise diagnostic criteria and advanced imaging techniques to ensure accurate differentiation, preventing misdiagnosis and its associated risks, such as sudden cardiac death (SCD). Understanding these adaptations and employing the appropriate imaging methods are crucial for athletes’ effective management and health optimization.

## 1. Introduction

Athlete’s heart (AH) is a condition that has garnered much interest and attention in the medical and general community and is characterized by a spectrum of structural, functional, and regulatory adaptations in the heart that arise due to prolonged and intensive athletic training [1,2,3]. An athlete is someone who regularly trains and participates in official sports competitions, irrespective of age or professional level [4]. AH changes typically include enlarged left and right cardiac chambers, increased left ventricular (LV) wall thickness, and cardiac mass [5,6]. Despite these changes, subjects maintain normal or enhanced heart function at rest and during exertion [5,6]. This remodeling shows the heart’s ability to adapt to the demands of high-intensity exercise [7].

Although athletes typically exhibit excellent health outcomes and increased longevity, significant physiological cardiac adaptations can overlap with subclinical manifestations of various pathological conditions in the so-called “grey zones” [8,9]. 

This overlap can obscure the distinction between benign adaptations and serious pathologies, some leading to sudden cardiac death (SCD) [1,10,11]. With more athletes and increased fitness interest, it is vital to distinguish between AH and structural abnormalities like dilated cardiomyopathy (DCM), hypertrophic cardiomyopathy (HCM), arrhythmogenic cardiomyopathy (ACM), and coronary artery anomalies [12,13,14]. Furthermore, accurate diagnosis is crucial as misdiagnosis can result in false reassurance, increased risk of SCD, delayed treatment, or incorrect exclusion from competitive sports [15]. Diagnosing AH primarily involves echocardiography, with cardiac magnetic resonance (CMR), computed tomography (CT), and nuclear scintigraphy as additional tools. Understanding physiological cardiac adaptations due to training is crucial [16,17]. Despite advancements in cardiac evaluation, the best imaging modality for AH remains debated [18]. This article reviews the imaging characteristics of AH and the roles of various modalities in distinguishing physiological adaptations from structural cardiac diseases.

## 2. Physiological Adaptations in the Athlete’s Heart

Research highlights that different exercise types result in different cardiac adaptation and hypertrophy patterns, influenced by changes in hemodynamic conditions, particularly cardiac output (CO) and systemic vascular resistance (SVR) [19,20,21]. Studies suggest that at least 3 h of training per week for at least three months could be sufficient to see some initial morpho-functional adaptations of the heart, but identifying an AH requires much more training [22]. Endurance training, which encompasses activities like running, cycling, swimming, and rowing [2,7], generally increases CO and stroke volume (SV) while maintaining normal or reduced SVR [21,23,24]. This type of training leads to an enlargement of heart chambers, including increased LV internal diameter and thickened LV walls (known as eccentric hypertrophy), accommodating high-volume loads [23,24,25]. In contrast, strength training involving activities such as weightlifting, bodybuilding, wrestling, and track-and-field throwing [2,7,26] is marked by short, intense repetitions that elevate SVR, leading to transient increases in systolic blood pressure while maintaining normal or slightly elevated CO [2,7,16]. The heart changes in this group are manifested by thickened LV walls without significant chamber dilation (so-called concentric hypertrophy), reflecting the heart’s adaptation to high resistance demands. In other words, both types have an increased LV mass index, but concentric hypertrophy features increased relative wall thickness (RWT), which is the ratio of wall thickness to diastolic ventricular diameter, and eccentric hypertrophy maintains normal RWT, resulting in a dilated LV with thinner walls due to increased myocyte length [27]. This sharp distinction between physiological cardiac adaptations is not always evident, as hybrid sports such as soccer, basketball, and volleyball incorporate endurance and strength training elements, leading to a blend of cardiac adaptations [2].

Moreover, these traditional differences have been challenged by recent studies suggesting that the increase in LV mass is proportional to the increase in LV volume (balanced remodeling) irrespective of the sports discipline, and normal LV geometry can frequently be observed in top-level athletes [28]. It should also be noted that heart size correlates with body size (frequently accounted for by adjustment for body surface area [BSA]), sex, and ethnicity, necessitating adjustments when comparing heart dimensions between individuals [18,29]. Typically, women have smaller hearts than men, reflecting differences in overall body stature [21].

## 3. Clinical and Electrocardiographic Evaluation

**Figure 1:**
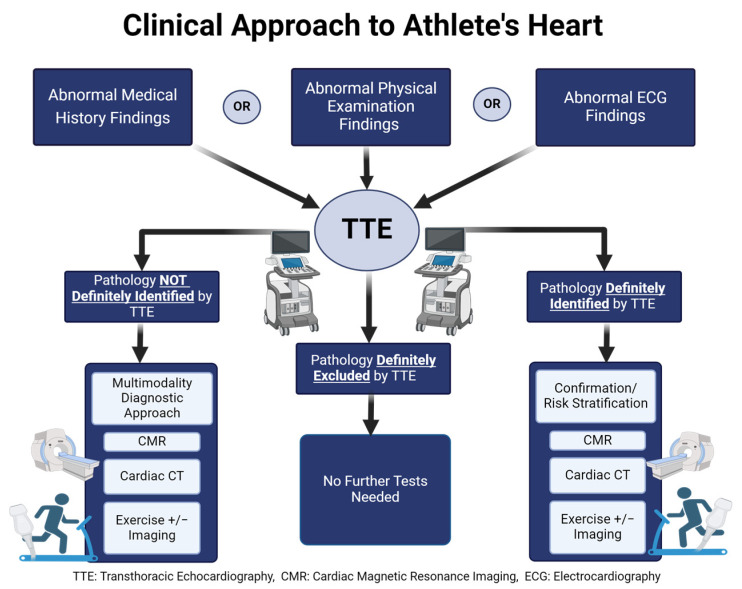
Clinical approach to the evaluation of athlete’s heart.

Cardiac evaluation in athletes is a structured and stepwise process designed to assess cardiovascular health and identify potential risks. SCD in young athletes is usually caused by genetic or congenital structural cardiac disorders such as HCM, ACM, or anomalous coronary artery origin. In athletes >35 years of age, most SCDs are due to atherosclerotic coronary artery disease (CAD) [30]. Therefore, pre-participation cardiovascular screening aims to identify pathological conditions in athletes to prevent morbidity and SCD and is recommended by international guidelines worldwide, even though the best strategies remain controversial [31]. A recent article proposed a systematic step-by-step approach to an athlete’s cardiovascular evaluation. It should begin with family and personal history collection, physical examination, and 12-lead resting ECG, as several scientific societies propose [32]. Notable increased risk findings in the medical history include a familial history of SCD or cardiomyopathies, as well as symptoms including chest discomfort, dyspnea out of proportion to the degree of exertion, palpitations, presyncope, and syncope. On physical examination, abnormal findings may include cardiac murmurs, paradoxical splitting of S2, and jugular venous abnormalities. Only in the presence of clinical suspicion or ECG abnormalities, it may be necessary to request other examinations, including cardiovascular imaging, as indicated in the International Recommendations for Electrocardiographic Interpretation in Athletes [33]. Most cardiovascular diseases in athletes can be suspected from an abnormal ECG [11,14,34,35]. Up to 80% of athletes exhibit benign ECG changes, including sinus bradycardia, first-degree AV block, and early repolarization [14,35]. These are typically linked to physical training and result from an increased vagal tone and/or decreased sympathetic activity [36]. Some ECG findings that require further investigation include T-wave inversion, ST-segment depression, pathologic Q-waves, complete left bundle branch block (LBBB), epsilon wave, ventricular pre-excitation, and prolonged QTc [34,35,36]. In that sense, the most common, accessible, and cost-effective exams as a second-line examination are echocardiography, exercise stress test (EST), 24 h Holter ECG monitoring, and cardiopulmonary exercise testing (CPET) [37]. If the results of one or more of these second-line evaluations are highly suspicious or fall in the grey zone, a third-line evaluation is needed, which is represented by less accessible or more costly diagnostic techniques such as exercise stress echocardiography (ESE), CMR, coronary computer tomography (CCTA), genetic testing, single-photon emission computed tomography (SPECT), and positron emission tomography (PET) [38] (Figure 1).

## 4. Cardiovascular Imaging in Athletes’ Heart

### 4.1. Echocardiography 

**Figure 2:**
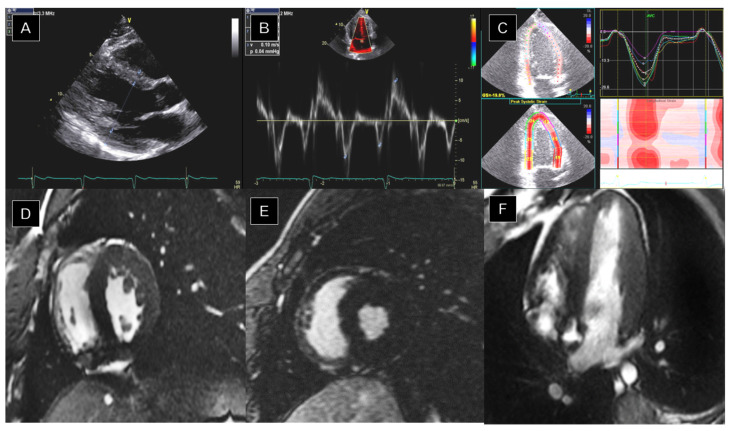
Transthoracic echocardiography (TTE) and cardiac magnetic resonance (CMR) from a patient with athlete’s heart. TTE showed left ventricular hypertrophy and borderline left ventricle diameters (**A**), maintaining normal diastolic (**B**) and systolic function with normal GLS (**C**). CMR confirmed the diagnosis of athletic adaptation, showing concentric left ventricular hypertrophy most marked in the mid-region of the lateral wall (19 mm), without LGE (**D**–**F**). Left ventricle ejection fraction EF is 69%. Notably, the wall thickness regressed with a period of detraining.

Transthoracic echocardiography (TTE) is the primary imaging tool for evaluating cardiac structure and function in athletes due to its accessibility, affordability, and noninvasive nature [26,29,39,40]. It offers insights into exercise-induced cardiac remodeling and comprehensively evaluates cardiac structures and function [29,39]. Furthermore, some authors suggested using a focused TTE in the pre-participation cardiovascular screening [41,42]. In recent years, progress in ultrasound technology has transformed the field of echocardiography. This test now provides detailed analyses of chamber measurements and systolic and diastolic function, using techniques such as 2D and 3D imaging and tissue Doppler imaging (TDI). It is invaluable in identifying “grey zone” athletes, where additional imaging modalities or longitudinal follow-up may be necessary (Figure 2) [4,43,44].

Doppler echocardiography has refined the understanding of cardiac remodeling, identifying normal adaptive changes from disease states [6,45]. LV myocardial strain measured by the speckle-tracking technique provides information on the deformation of the myocardium [5,6,46]. However, TTE also has limitations, including operator dependency and the impact of body size and anatomy on achieving clear views, which can affect data interpretations in the evaluation of AH and lead to the need for more sophisticated studies (Table 1 and Table 2) [47]. Several reference values about age, gender, ethnicity, and sports disciplines have been published in the literature by different study groups. However, we currently lack universally accepted cut-offs for basic echocardiographic measurements, and therefore, there are no unanimous recommendations about using echocardiographic cut-offs to distinguish between physiological and pathological adaptations [32]. Despite these limitations, TTE remains a valuable and front-line tool in the screening of athletes who are flagged as being at risk of cardiac pathology [46]. 

#### 4.1.1. Left Ventricle (LV) Dimensions, Mass, and Wall Thickness

Long-term athletic activities have been shown to impact the size, wall thickness, and function of the LV [13,19,20,23,24,47,48,49]. Many studies have established reference values based on factors like age, gender, ethnicity, and types of physical activity. Using echocardiography to measure LV dimensions, Pellicia et al. studied 1309 elite Italian athletes across 38 sports. The authors found LV end-diastolic dimensions ranging from 38 to 70 mm, with 55% of athletes within the normal range (≤54 mm). While 14% exhibited significant enlargement (over 60 mm), they maintained normal LV systolic function, had no regional wall-motion abnormalities, and were symptom-free. The authors showcased that cavity size mainly correlates with body size and participation in endurance sports such as cycling and skiing, consistent with findings from other studies [50]. Utomi et al. conducted a large meta-analysis, including 185 echocardiographic and 41 MRI datasets, comparing cardiac structures between athletes and sedentary controls. Findings showed that endurance and strength-trained athletes have larger LV dimensions than sedentary controls, with more pronounced differences in endurance athletes, especially cyclists and rowers [47]. Endurance athletes had notably larger left ventricular end-diastolic diameters (LVEDDs) and volumes (LVEDVs) than strength athletes but similar thicknesses in the interventricular septum and posterior wall. Another meta-analysis by Pluim et al. examined echocardiographic data on cardiac structures in different athlete groups (endurance, strength, and combined) and control subjects. They found no significant differences in LV mass between the groups [51] (Table 3).

#### 4.1.2. Left Ventricular Systolic Function

Prior studies show that athletes maintain normal LV systolic and diastolic functions and ejection fraction (EF) comparable to non-active controls [46,47,48]. In elite cyclists, LV ejection fraction could be slightly reduced, but global LV systolic function was within the normal range [43,44]. This reduction in EF, partly due to altered cardiac loading, highlights a “grey zone” in distinguishing early DCM from AH, especially with EF below 55%. LV strain and strain rate imaging, via TDI or speckle-tracking echocardiography, may help differentiate maladaptive from physiological remodeling [6,45]. Speckle-tracking echocardiography measures heart muscle movement and deformation, providing detailed regional and global LV function analysis by tracking speckle movement frame-by-frame [46]. TDI measures the velocity of myocardial movements using Doppler principles. It evaluates the speed at which the myocardium contracts and relaxes [47]. Strain imaging, via TDI or speckle-tracking echocardiography, measures the percentage change in myocardial fiber length during the cardiac cycle. Left ventricular global longitudinal strain (LV GLS) is considered more sensitive than LVEF for identifying subclinical LV dysfunction (Table 4) [32,48]. A meta-analysis conducted to assess the variability in GLS in a normal population included 16 papers from 2011 onwards. The mean GLS was found to be 20.7% [19.2, 22.7], with variations influenced by age, weight, systolic blood pressure, and vendor. GLS < 16% was found in 2.8% of participants, indicating significant myocardial dysfunction [49]. Meta-analyses and systematic reviews present mixed results on the normal values of GLS in AH. Some studies report higher GLS in athletes than non-athletes, while others find no difference or report GLS values on the lower side of the normal range in athletes. This variability in GLS is likely due to differences in training type, volume, and left ventricular structure but can also be influenced by the method’s variability as mentioned above [50]. A study involving 425 healthy, competitive elite athletes, compared to an age- and sex-matched sedentary control group, investigated the impact of training on cardiac morphology and function. Findings revealed that athletes had normal but lower resting LV GLS values compared to sedentary controls. Even when this difference showed statistical significance, it did not imply clinical relevance.

Power athletes had the lowest resting LV GLS values compared to mixed and endurance athletes due to static exercise regimes imposing a significant pressure load on the LV [51]. Resting LV mechanics in athletes are characterized by a balanced decrease in GLS and global circumferential strain (GCS). A balanced decrease in LV GLS and GCS suggests a physiological rather than pathological process [51]. One research study including 200 Olympic athletes and 50 controls used two-dimensional speckle-tracking echocardiography to evaluate LV myocardial mechanics. Participants aged 15–40, included male and female athletes in various disciplines. Athletes had normal but slightly lower GLS values than controls, with endurance athletes having the lowest [51]. Another study compared 87 professional athletes (37 cyclists, 29 soccer players, and 21 handball players) with 125 patients having various LVH forms (HCM, hypertensive heart disease, and severe aortic valve stenosis); 37 untrained individuals served as controls. GLS was reduced in patients with pathological hypertrophy compared with athletes and controls [50]. Further research is essential to determine and standardize GLS values across different athlete populations, including variations in ethnicity, gender, and types of exercise. 

Myocardial work (MW), a new index for cardiac function, is less load-dependent than GLS and calculated using LV strain and noninvasive LV pressure. A study of 250 strength athletes (62% male) and 180 controls used echocardiography and LV 2D speckle-tracking strain at rest and during exercise. Results showed comparable LVEF but reduced LV GLS in athletes. MW did not differ significantly between groups. Athletes had superior exercise capacity, peak VO_2_, and higher pulmonary artery systolic pressure (PASP) at peak exercise. MW at rest predicted exercise capacity, peak VO_2_, and PASP [62].

#### 4.1.3. Left Ventricular Diastolic Function

Research shows mixed results on athletes’ diastolic function. Utomi et al. found no significant difference in peak E velocity, suggesting similar early diastolic filling in athletes and sedentary controls [47]. Researchers observed a higher E/A ratio and increased early myocardial mitral tissue velocities (E’) in endurance athletes, indicating enhanced early diastolic filling due to extended training [63]. Brown et al. noted decreased E and e’ velocities in elite cyclists compared to recreational ones, suggesting elite athletes have significant functional reserves for high performance under extreme demands [64]. Further research is needed to determine if this pattern is consistent among elite athletes in various sports. George et al. and Dores et al. found that despite cardiac remodeling, athletes’ E/A ratios remained normal and higher than non-athletes due to A wave reduced velocity, indicating training adaptation, not pathology [10,65]. These findings highlight the complexity of adaptations in AH and the need for further research into the cardiovascular effects of high-level athletic training.

#### 4.1.4. Right Ventricle 

Exercise training can result in changes to the right ventricle (RV) function and structural remodeling, but research on the RV adaptations to high-intensity training has traditionally been less advanced than for the LV [1,2]. The complex anatomy of RV makes it challenging for conventional echocardiography to assess its size and function accurately [66]. The RV shares the hemodynamic load with the LV and responds to intensive exercise similarly, increasing in size while maintaining systolic and diastolic function [29]. Studies by Baggish et al. and D’Andrea et al. demonstrate that elite athletes, particularly endurance athletes, have larger RV dimensions and better systolic function than less trained or sedentary individuals [6,26]. They typically display increased RV end-diastolic volume (RVEDV), increased wall thickness, RV mass, and RV stroke volume (RVSV) compared to controls (Table 5). Long-term endurance training promotes an increase in all RV-indexed dimensions and induces a more spherical RV shape in both male and female athletes [43].

#### 4.1.5. Atria

The left and right Atria undergo changes in AH, observed in echocardiograms [68]. Most studies indicate that increased atrial dimensions in athletes can revert to normal following a period of detraining. Assessing the left atrial (LA) function requires the evaluation of multiple parameters that reflect its reservoir, conduit, and contraction functions. While some research shows that LA function is preserved despite increased volume, other studies have observed a reduction in function following intensive training. Studies have shown that LA remodeling in athletes is characterized by increased LA volume index (LAVI) and improved myocardial diastolic properties, which can be identified using DTI and speckle-tracking echocardiography [6,69]. 

Strain imaging enhances understanding of LA mechanics. Reference values for peak longitudinal LA strain (PALS) and peak atrial contraction strain in healthy subjects are 45.5% ± 11.4% and 2.11 ± 0.61 s^−1^, respectively, varying based on the cardiac cycle phase [70,71]. A meta-analysis of nine studies involving 403 elite athletes and 297 controls revealed higher LA volumes in athletes but reduced global LA longitudinal strain (PALS), indicating decreased reservoir function [71]. Late diastolic LA strain rate (LASRlate) is also lower in athletes, while contraction strain and systolic strain (LASRsyst) are similar between athletes and controls. The meta-analysis suggests that reduced LA function in athletes may contribute to a higher risk of atrial fibrillation. A study of 1777 competitive athletes, predominantly male and free from structural cardiovascular disease, explored LA size and its correlation with propensity for supraventricular arrhythmias. LA enlargement (≥40 mm) was observed in 20% of athletes, with 2% showing marked dilation (≥45 mm). Supraventricular tachyarrhythmias were rare (<1%), and occurrence rates in athletes were like non-athletes, irrespective of LA enlargement [72]. LA enlargement was linked to LV cavity size and engagement in dynamic sports but not to body size. The study concluded that LA enlargement in highly trained athletes is generally a physiological response to exercise and is not associated with adverse clinical outcomes [72]. Although LA enlargement is typically associated with an increased risk of atrial fibrillation (AF), AF is only more prevalent in endurance athletes, especially men and those who begin competing at a young age. However, data on AF in female athletes and ethnic minorities are lacking [61]. Differentiating between physiological and pathological LA remodeling using combined volumetric and strain evaluations is crucial. Despite limitations, echocardiography and CMR remain valuable for assessing LA function and potential risks in athletes.

#### 4.1.6. Aorta

Aortic dilatation, a principal risk factor for acute aortic syndromes, is frequently encountered among endurance athletes, but its prevalence, implications, and association with exercise are not well understood [73,74]. A cross-sectional study assessed 442 masters-level endurance athletes (aged 50–75 years), including rowers and runners, using TTE to measure aortic sizes at the sinuses of Valsalva and the ascending aorta. Compared to age, sex, and body size-adjusted aortic dimensions, 21% of athletes had an ascending aortic size of 40 mm or larger, with 31% of men and 6% of women meeting this criterion. Larger aortic dimensions were associated with elite competitor status, particularly in rowing, as well as age, sex, body size, and hypertension. The study concluded that aortic dilatation is common among aging endurance athletes, suggesting long-term exercise may lead to vascular remodeling like cardiac remodeling. This highlights the need for follow-up to determine clinical outcomes and the risk of acute aortic syndromes [75,76].

### 4.2. Exercise Stress Echocardiography (ESE)

ESE is a reliable, safe, noninvasive imaging test that evaluates dynamic cardiac function. ESE’s primary importance lies in detecting exercise-induced abnormalities, such as ischemia and arrhythmias, which might not be visible at rest in athletes with suspected coronary artery disease or congenital coronary artery anomalies [4,77]. Furthermore, ESE can assess contractile reserve during exercise in endurance athletes with LV and/or RV dilatation and mildly reduced EF at rest, and an increase of LV EF of at least 15% during exercise may support the diagnosis of AH [32]. Finally, ESE may be useful in athletes with valvular heart disease, providing information about exercise tolerance, biventricular contractile reserve, changes in hemodynamics (LV filling pressure and pulmonary pressure), and valvular functional parameters (transvalvular gradients and regurgitation entity—i.e., bicuspid aortic valve) [77]. The noninvasive nature and ability to simulate physiological exercise conditions make ESE preferable over pharmacological stress testing for physically capable athletes [78]. Despite limitations like potential false positives and the need for specialized equipment, ESE remains indispensable in sports cardiology for ensuring accurate diagnosis and management of cardiovascular health in athletes.

### 4.3. Cardiac Magnetic Resonance (CMR) Imaging 

CMR is a valuable diagnostic modality to distinguish normal adaptation from cardiomyopathy and aids in risk stratification by accurately characterizing myocardial volumes, mass, contractility, and wall motion [74]. CMR is the gold standard for defining biventricular volumes and mass and quantifying volumes and flow, providing advanced myocardial tissue characterization with excellent accuracy and precision [79]. 

CMR is particularly useful in cases where echocardiography results are uncertain [80,81]. However, its use as a primary screening tool is limited due to availability and cost, the potential for false positives, and the lack of prognostic significance for certain findings [82]. CMR enables accurate morphological and functional assessment, including tissue characterization by late gadolinium enhancement (LGE), which can reveal distinctive patterns in HCM and other myocardial conditions [83,84]. CMR can detect replacement fibrosis, edema, and fat within the myocardial walls [84,85,86].

Mild myocardial fibrosis is more common in athletes than non-athletes, usually involving less than 3% of the myocardium. It varies in quantity, location, and pattern and is often found in the RV or interventricular septum, especially in endurance athletes. The prognostic significance of myocardial fibrosis in athletes remains unclear [87]. T2 mapping identifies active myocardial edema or inflammation, showing normal values in strength-trained athletes but elevated values in early DCM and HCM [88]. Lastly, the potential of CMR-based deformation imaging to differentiate between AH and pathological phenotypes is currently being studied in various disease settings. This aspect of CMR could enhance diagnostic capabilities by offering detailed insights into myocardial deformation patterns unique to physiological adaptations or disease processes [76,89]. CMR is essential for assessing RV changes in athletes, providing detailed imaging to distinguish physiologic adaptations from conditions like ACM. The CMR criteria for ACM include major criteria such as regional RV dyskinesia, akinesia, or aneurysm, severe RV dilatation, and reduced RV EF (RVEDV index >110 mL/m² for males, >100 mL/m² for females; RV EF < 40%). Minor criteria include mild or moderate RV dilatation and/or reduced RV EF (RVEDV index 100–110 mL/m² for males, 90–100 mL/m² for females; RV EF 40–45%) and regional RV dyskinesia or akinesia. These criteria are part of the revised Task Force criteria for diagnosing ACM [90]. 

While CMR offers numerous benefits, its limitations include limited availability and higher costs than echocardiography. This makes it less accessible, especially in resource-limited settings. Patient factors, such as underlying renal disease, can limit its use due to the risks of gadolinium-based contrast agents. Additionally, the enclosed space of the equipment can provoke claustrophobia in some patients. These factors add complexity to choosing CMR for first-line cardiac evaluation despite its superior diagnostic capabilities. Stress CMR, performed with exercise, is another valuable tool. It can detect reduced functional reserve and early-stage cardiomyopathy even when resting assessments show only mild abnormalities. However, its accessibility is limited due to the need for CMR-compatible equipment, and the cost-effectiveness of using stress CMR in this context still requires further evaluation [91]. 

### 4.4. Computed Tomography (CT) Imaging 

Cardiac computed tomography angiography (CCTA) offers a precise, noninvasive method to assess coronary artery anatomy and great vessel dimensions. Despite its accuracy, there is a paucity of studies dedicated to its application in athletes, resulting in CCTA not being commonly utilized as the primary imaging modality for AH. Nonetheless, when there are suspicions of anomalous coronary arteries or aortopathy, both CCTA and CMR can provide detailed anatomical evaluations [39,92]. Furthermore, when dilatation of the aortic root or ascending aorta is suspected, at least one comprehensive aortic tomographic assessment by CCTA should be performed [26]. Cardiac CT imaging with ECG gating accurately assesses coronary artery disease and atherosclerosis [93] but is limited by radiation exposure and cost, making it less suitable for young athletes [5]. Coronary artery calcification (CAC) is more prevalent in athletes, particularly those engaged in high-intensity exercise. Studies, including the MARC-2 study, have found that athletes have higher rates of CAC compared to non-athletes [94]. The increased calcification is associated with higher levels of exercise over a person’s lifetime, as well as the intensity of the exercise. The calcified plaques in athletes are typically more stable, which reduces the likelihood of rupture and subsequent acute cardiovascular events, explaining why athletes may have higher CAC but fewer heart attacks. The MARC-2 study showed that while the volume of exercise did not correlate with CAC progression, the intensity of exercise did. Vigorous exercise was linked to less CAC progression, whereas very vigorous exercise was associated with greater progression of both CAC and calcified plaques. The increased coronary calcification in athletes is thought to result from the heightened mechanical stress, increased heart rate, and elevated catecholamine levels associated with high-intensity exercise. Overall, despite the higher prevalence of CAC, athletes generally have a lower cardiovascular risk profile due to protective factors such as better cholesterol levels and superior cardiorespiratory fitness. This can mitigate the clinical impact of the calcification [94].

### 4.5. Nuclear Imaging Techniques

Research on myocardial perfusion imaging (MPI) in AH is limited. Nuclear imaging, including MPI with SPECT and PET, can detect myocardial ischemia [95,96]. Nuclear imaging can rule out ischemia where suspected and aid in distinguishing between HCM and athlete’s heart. PET can evaluate myocardial blood flow and flow reserve [97]. Research indicates that while baseline myocardial blood flow in HCM resembles that of normal controls, the response to maximal vasodilation is reduced, highlighting impaired myocardial blood flow in these patients [98]. However, PET may be preferred in balanced 3-vessel disease since it permits absolute quantification of myocardial blood flow. Conversely, SPECT can only provide semi-quantitative values (normalized to the maximum value), failing to detect relative perfusion differences [99]. Moreover, even SPECT specificity in competitive athletes has to be considered reduced, given that myocardial perfusion defects can be present also in healthy young male athletes, and they are associated with LV hypertrophy and no wall motion abnormalities on echocardiography [100]. Thus, cardiac nuclear imaging in the athlete’s setting is more suitable for research purposes than for a clinical application and should not be recommended as a first-line test in competitive athletes.

## 5. Imaging Modalities in Differentiating Athlete’s Heart from Cardiovascular Diseases

**Figure 3:**
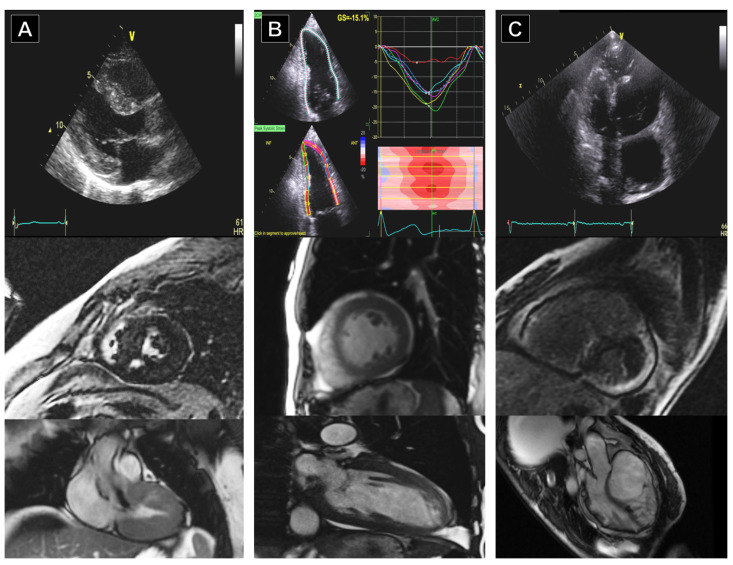
Patient 1 (**A**): A patient with left ventricular hypertrophy in transthoracic echocardiography who exhibits delayed gadolinium enhancement in the region of the anterior septum on CMR. Patchy enhancement is focal, with another tiny enhancement seen along the base in the mid-septum, compatible with HCM. Patient 2 (**B**): A patient with increased left ventricle diameters and mildly reduced systolic function in echocardiography. CMR findings were consistent with dilated, nonischemic cardiomyopathy, confirming the left ventricle dilatation and decreased global systolic dysfunction. Patient 3 (**C**): A patient with ARVC who showed severe right ventricle and right atria dilation with diminished right ventricle function. Extensive sacculation, right ventricle hypertrophy, and enlargement are consistent with the diagnosis of ARVC. The extensive LGE along the right ventricle free wall and right-sided interventricular septum further support this diagnosis.

**Figure 4:**
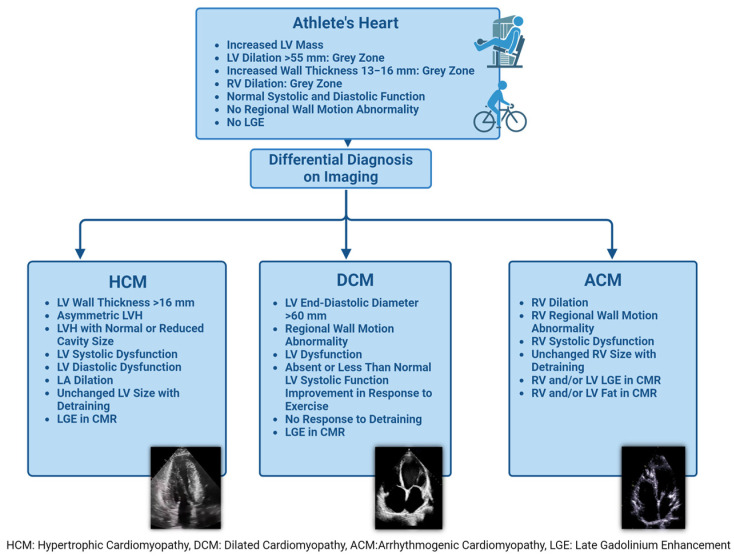
Imaging findings in athlete’s heart and important differential cardiomyopathies.

It is necessary to have a precise definition of the features of the AH and stringent criteria to optimize the clinical management of athletic subjects to be able to make a differential diagnosis with HCM, DCM, LVNC, and ACM [101] (Figure 3 and Figure 4). It is crucial to emphasize that the effective use of clinical imaging data must be integrated with other clinical aspects, such as the presence or absence of symptoms, family history of genetic heart disease or SCD, and 12-lead ECG results. Therefore, the choice of diagnostic approach should always be guided by clinical suspicions and consider the entire clinical scenario, encompassing the full spectrum of cardiovascular diseases that can affect athletes [32]. 

### 5.1. Dilated Cardiomyopathy (DCM)

Increased LVEDDs represent a “grey zone” of clinical uncertainty, and an LVEDD greater than 60 mm may indicate DCM rather than physiologic adaptation to athleticism [102,103]. Despite often having severely enlarged LV dimensions, athletes typically maintain a preserved or low-normal EF, normal wall motion, and superior diastolic performance, differentiating their condition from DCM [102,104]. One study included 35 males with DCM, 25 athletes in the grey zone (defined as an athlete with LV enlargement and borderline EF < 55%), and 24 athletes with normal EF, who underwent various cardiac tests. ESE showed a marked increase in EF during exercise for grey-zone athletes, differentiating them from DCM patients. Athletes also exhibited enhanced LV diastolic function and higher medial annular tissue Doppler velocities, aiding in swift LV relaxation and SV maintenance during high heart rates [78]. Endurance athletes had higher LV early diastolic tissue velocities than controls, indicating better diastolic function. 

Additionally, a significant reduction in LV dimensions after three months of detraining can help in differentiate AH from DCM [74,105]. Moreover, ESE, an excellent, noninvasive test for athletes, can detect exercise-induced ischemia and assess contractile reserve in athletes with ventricular dilatation and mildly reduced LVEF to help differentiate them from DCM [106]. Millar et al. found that only 17% of individuals with DCM could reach a peak exercise LVEF over 63%, compared to 92% of grey-zone athletes and controls. Combining the criteria of an increase in LVEF by more than 11% and a peak LVEF exceeding 63% achieved 86% sensitivity and 92% specificity in identifying impaired contractile reserve [78].

### 5.2. Hypertrophic Cardiomyopathy (HCM)

Although LV wall thickness typically remains within normal limits in athletes, challenges arise when it measures between 13 and 16 mm, especially if asymmetric, potentially overlapping with mild cases of HCM [47,51,82,103,107]. Left ventricular hypertrophy (LVH) diagnosis requires calculating the LV mass index and is assessed by measuring the thickness of the LV wall and septum [5,108,109]. Sharma et al. investigated LVH in elite adolescent athletes to differentiate it from HCM. This study showed that athletes had greater left ventricular wall thickness (LVWT) than sedentary controls. Very few athletes had LVWT exceeding 12 mm, and ventricular enlargement was consistently observed in such cases. LVWT > 12 mm without ventricular dilation in male adolescent athletes should prompt consideration of HCM [110]. Normal or increased LVEDD, systolic/diastolic function, and atrial size can help assess whether hypertrophy is reversible and exercise-induced [109,111]. 

There is controversial data regarding detraining and subsequent improvement in cardiac imaging to differentiate AH from HCM. A study on strength-trained athletes with concentric hypertrophy found significant reductions in LV mass and wall thickness after detraining, indicating exercise as the hypertrophy’s origin [111]. However, another study noted that significant regression of LVH following detraining can occur in HCM, and this phenomenon is not exclusive to an athlete’s heart [112].

Speckle-tracking echocardiography and CMR are also useful for differentiation. Speckle tracking analyzes LV twisting and untwisting to distinguish AH from HCM. In HCM, CMR shows myocardial fibrosis, often mid-wall and in areas of maximum hypertrophy, serving as a key pathology indicator, whereas such fibrosis is generally absent in AH [83,87].

### 5.3. Arrhythmogenic Cardiomyopathy (ACM)

One of the main challenges lies in distinguishing RV physiological adaptations from pathological conditions such as ACM, especially in those cases that fall within the “grey zone” of uncertainty. ACM is an inheritable heart disease caused by dysfunctional cardiac desmosomes, leading to fibrofatty replacement and structural and functional alterations of the ventricles [113]. The diagnosis of ACM should follow the 2010 Task Force criteria, which include electrical parameters, imaging, tissue properties, family history, and genetic testing [114]. RV size is a key diagnostic parameter for ACM but not AH, often leading to false suspicions. Echocardiography of elite athletes revealed that RV and right atrial dimensions are greater in endurance-trained athletes compared to age- and sex-matched strength-trained athletes and sedentary controls. However, other typical findings of ACM, such as RV bulging, thinning, and aneurysms, are usually absent in healthy athletes [115]. D’Ascenzi et al. assessed RV remodeling in Olympic athletes, particularly males and those in endurance sports. Using two-dimensional echocardiography and Doppler imaging, they found that many highly trained athletes have RV dimensions exceeding conventional clinical reference limits but do not exhibit signs of RV dysfunction. Therefore, the researchers recommended using the upper limits (95th percentile) of RV dimensions measured in their cohort of Olympic athletes as new reference values for assessing RV size in highly trained athletes. This means that RV dimensions should be considered normal if they fall within the range observed in 95% of the athletes in their study. Alternatively, they suggested using only the major criteria defined by the Task Force criteria for diagnosing ACM to avoid misclassifying normal athletic remodeling as pathological [24]. Among all imaging modalities, CMR is the preferred method for evaluating the RV. It can detect RV enlargement and identify other pathological features of ACM, such as wall motion abnormalities, RV systolic dysfunction, RV fibrofatty replacement, and sub-epicardial and mid-wall LGE, indicating fibrosis [116,117]. 

### 5.4. Left Ventricular Non-Compaction (LVNC)

Left ventricular non-compaction (LVNC) is a cardiomyopathy characterized by increased trabeculation and deep intertrabecular recesses within the LV. It is associated with progressive LV dilatation, impaired systolic and diastolic function, life-threatening arrhythmias, and thromboembolic events [118]. Hypertrabeculation can also occur as a physiological adaptation to the increased loading conditions of intense exercise. The distinction between “hypertrabeculation”, a potential physiological adaptation to intensive training, and “LVNC,” a cardiomyopathy associated with life-threatening arrhythmias, LV dilatation, and dysfunction, is essential. In a case series of four professional basketball players [119], marked hypertrabeculation was observed after a period of intensive training. This condition peaked during the most intensive conditioning phase and decreased after a period of detraining. Importantly, LV systolic and diastolic functions remained normal despite the increased trabeculation, suggesting that the hypertrabeculation was a benign adaptation rather than a pathological condition [119]. Considering family history, symptoms, and the absence of doping drugs is crucial when evaluating athletes for LVNC. 

Further, longitudinal studies are needed to better understand the long-term effects of exercise training on LV morphology and function in athletes, given the limited prospective data and the risk of overdiagnosis in low-risk populations.

## 6. Artificial Intelligence in Evaluating Athlete’s Heart

Artificial intelligence (AI) offers significant advantages in assessing the AH by enhancing risk assessment and diagnostic accuracy, improving early detection and prevention, and providing consistent, automated interpretations of diagnostic tests. It integrates data from various sources, enabling a holistic view and personalized treatment plans. AI’s real-time monitoring through wearables allows for immediate feedback and timely interventions. Additionally, AI saves time for healthcare professionals and can predict potential health issues, improving overall cardiovascular care for athletes [120,121]. 

AI algorithms, including deep learning (DL) techniques, are likely to soon assist with challenging aspects of athletes’ cardiovascular imaging, especially echocardiography [120,122]. They can identify unique patterns and aid in the differential diagnosis of serious conditions [122,123]. A study that combined speckle-tracking echocardiography with machine learning (ML) demonstrated high sensitivity and specificity in distinguishing between HCM and AH [124]. Other studies have shown that DL algorithms outperformed echocardiography specialists in diagnosing causes of LVH [59]. ML integrated with CMR for measurements has surpassed human experts, potentially enhancing diagnostic accuracy and risk assessment [125]. AI innovations also improved image acquisition, reconstruction, quality, and analysis. 

Bernardino et al. conducted a study using a linear statistical shape analysis framework to examine cardiac remodeling in athletes [126]. Statistical shape analysis helps to study organ shapes and identify disease-related changes. However, confounding factors, like age and body mass index, can distort the results if not properly addressed. The presented framework includes methods to correct for these confounders. The framework was applied to cardiac MRI data of 89 triathletes and 77 controls to study cardiac remodeling due to endurance exercise. Their methodology helped visually illustrate key shape differences, effectively highlighting areas of cardiac remodeling in athletes through CMR imaging. The potential for utilizing AI and ML in diagnosing CV conditions in athletes is likely to significantly transform practice, with a substantial amount of ongoing research and investigation in this area [103].

## 7. Conclusions

The evaluation and understanding of AH have advanced significantly with various imaging modalities, each essential for distinguishing between physiological adaptations and potential cardiac pathologies. The “gray zone” continues to present clinical challenges, often necessitating additional imaging techniques or extended follow-up for a definitive diagnosis. When assessing competitive athletes, it is vital to consider factors such as demographic data, ECG results, and symptoms. While echocardiography will likely remain the primary imaging method, CMR provides deeper insights in more ambiguous cases. Advances in AI and ML show promise for improving screening, diagnostic accuracy, and personalized cardiovascular assessments for athletes. However, continued research and interdisciplinary collaboration are crucial to deepen our understanding of cardiovascular adaptations to long-term physical training, with comprehensive assessments being essential for differentiating AH from pathological cardiomyopathies.

## Figures and Tables

**Table 1:** Common imaging diagnostic modalities for cardiac evaluation in athletes.

Modalities	Advantages	Limitations
Echocardiography	- Widely available and inexpensive. - Good assessment of myocardial wall thickness; cavity size; chamber function, including EF and strain, valvular abnormalities; and diastolic function. - No radiation exposure.	- Image quality can be challenging in some cases, inter-observer variability, challenges in evaluating the right ventricle (RV), pulsed wave doppler has angle dependency, and Doppler tissue imaging has poor spatial resolution.
Cardiac Magnetic Resonance	- Detailed characterization of the myocardium with high spatial resolution, including presence of scar. - The gold standard for assessing myocardial wall thickness, cavity size, systolic function, and RV. - Demonstrates coronary artery origins, no radiation.	Limited availability, being relatively expensive, logistics, claustrophobic patients, and time-consuming data acquisition and analysis.
Cardiac Computed Tomography	High spatial resolution in evaluating coronary atherosclerosis and precise delineation of coronary origins and pathways.	Exposure to ionizing radiation, cost, low temporal resolution, and dependent on renal function.

**Table 2:** Comparison among imaging diagnostic modalities for cardiac evaluation in athletes.

	2D Echo	CMR	CT	SPECT	PET
LV volumes and function	+++	++++	+++	++	++
Valvular disease	++++	+++	+	—	—
Ischemia/perfusion	+++	+++	+	+++	++++
Morphology of the coronary arteries	—	++	+++	—	—
Imaging fibrosis	—	++++	++	—	++
Spatial resolution	+++	+++	+++	++	++
Temporal resolution	++++	++	+	++	++
Limitations	Operator dependence, acoustic window	Availability, incompatible devices, renal failure	Availability, radiation, renal failure	Availability, radiation	Availability, radiation

CMR: Cardiac magnetic resonance; CT: computed tomography; PET: positron emission tomography; SPECT: single-photon emission computed tomography; “—” indicates no role in diagnosis, while “+” shows the tool has a role. More “+” symbols signify greater importance.

**Table 3:** Left heart chamber echocardiography evaluation in athlete’s heart.

Parameter	Athlete Gender	Exercise Type	General Population [52,53]
LV mass (g) [47,48]	Female: 143.9 ± 31.4	Endurance: 232 (200–260)	Female: 66–150
	Male: 207.8 ± 47.0	Strength: 220 (205–234)	Male: 96–200
LVEDD (mm) [51]	Female: 48.8 ± 3.3	Endurance: 53.7 (52.8–54.6)	Female: 45.0 ± 3.6
	Male: 54.1 ± 3.8	Strength: 52.1 (50.6–53.6)	Male: 50.2 ± 4.1
LV IVS (mm) [47,48]	Female: 8.7 ± 0.8	Endurance: 10.6 (10.3 to 10.9)	Female: 6–9
	Male: 10.1 ± 1.0	Strength: 10.4 (9.8 to 10.9)	Male: 6–10
LV PWT (mm) [51,54,55]	Female: 8.4 ± 0.8	Endurance: 9.7 ± 3.1	Female: 6–9
	Male: 9.8 ± 0.5	Strength: 11.3 ± 2.4	Male: 6–10
Relative Wall Thickness [55]	- -	Endurance: 0.37 ± 0.04	Female: 0.22–0.42
		Strength: 0.45 ± 0.06	Male: 0.24–0.42
LV Systolic Function (EF%) [48,56]	Female: 61.7 ± 3.7	Endurance: 66 ± 5%	Female: 54–74
	Male: 63.2 ± 3.5	Strength: 67 ± 6%	Male: 52–72
LV Diastolic Function (E/A) (cm/s) [57]	Female: 1.94 ± 0.49	Endurance: 2.02 ± 0.51	Female: ≥0.8
	Male: 1.92 ± 0.51	Strength: 1.83 ± 0.48	Male: ≥0.8
DT (ms) [58]	- -	Endurance: 141 ± 15.8	150–240
		Strength: 155 ± 14.4	
LA diameter (cm) [52,59]	Female: 3.0 ±0.4	Endurance: 3.86–4.08	Female: 2.7–3.8
	Male: 3.4 ± 0.4	Strength: 3.63–3.87	Male: 3.0–4.0
LA Area (cm²) [60]	- -	20.7 ± 4.4	Female: ≤20
			Male: ≤20
LA Volume Index (mL/m²) [55]	- -	Endurance: 29.1 ± 9.1	Female: 16–34
		Strength: 26.4 ± 8.4	Male: 16–34

**Table 4:** Left ventricular strain rate imaging variables [61].

Variable	Endurance Athletes	Strength Athletes
Basal Segment Strain Rate (SR, s^−^¹)	−0.8 ± 0.3	−1.4 ± 0.4
Mid Segment Strain Rate (SR, s^−^¹)	−1.0 ± 0.4	−1.3 ± 0.3
Apical Segment Strain Rate (SR, s^−^¹)	−1.1 ± 0.3	−1.5 ± 0.1
Mean Strain Rate of 16 Segments (SR, s^−^¹)	−1.0 ± 0.4	−1.4 ± 0.4

**Table 5:** Right heart chambers echocardiography evaluation in athlete’s heart.

Parameter	Athlete Gender	Exercise Type	General Population [58,67]
RV wall thickness (mm) [24]	-	Male Strength: 4.0 (3.5)	≤5 mm
	-	Male Endurance: 4.2 (3.9–4.4)	
RV end-diastolic area (cm²) [58]	Female: 21.4 ± 2.7	Endurance: 26.6 ± 4.0	11.5–18.8
	Male: 24.1 ± 3.1	Strength: 22.0 ± 3.5	
RV end-systolic area (cm²) [58]	Female: 11.2 ± 1.9	Endurance: 13.2 ± 2.7	6.3–12.2
	Male: 12.9 ± 2.2	Strength: 10.5 ± 1.5	
TAPSE (mm) [24]	Female: 39 ± 4	Male Strength: 41 (32–49)	17–23
	Male: -	Male Endurance: 35 (32–38)	
RV FAC (%) [58]	- -	Strength: 51.2 ± 9.8	35–45%
		Endurance: 50.4 ± 7.0	
RA area (cm²) [58]	- -	Strength: 15.0 ± 3.6	<18
		Endurance: 17.5 ± 2.7	
